# A randomized controlled trial for gualou danshen granules in the treatment of unstable angina pectoris patients with phlegm-blood stasis syndrome

**DOI:** 10.1097/MD.0000000000021593

**Published:** 2020-08-14

**Authors:** Jianbo Guo, Shuang Dai, Yukun Ding, Haoqiang He, Hui Zhang, Wenchao Dan, Kun Qin, Hui Wang, Anqi Li, Peipei Meng, Shangjin Li, Qingyong He

**Affiliations:** a Department of Cardiology, Guang’anmen Hospital, China Academy of Chinese Medical Sciences, Beijing 100053, China; b Beijing University of Chinese Medicine, Beijing 100029, China; c Henan University of Chinese Medicine, Henan 450000, China.

**Keywords:** Gualou Danshen granules, phlegm-blood stasis syndrome, unstable angina pectoris

## Abstract

**Introduction:**

Unstable angina pectoris is an acute exacerbation secondary to coronary artery occlusion. In routine clinical treatment, patients with unstable angina pectoris are prone to recurrence or aggravation of symptoms. Based on the traditional Chinese medicine (TCM) theory, phlegm, and blood stasis are one of the main pathological factors of unstable angina pectoris. The treatment of unstable angina pectoris with phlegm-blood stasis syndrome by Gualou Danshen granules (GLDS) has been the focus of many clinical trials. However, there is no evidence to prove the safety or clinical efficacy of GLDS.

**Methods and analysis:**

In this study, we will conduct a 4-week randomized, controlled feasibility study, with participants recruited from Guang’anmen Hospital, Chinese Academy of Traditional Chinese Medicine. Sixty subjects are to be diagnosed as having phlegm-blood stasis syndrome and randomly divided into a treatment group (GLDS) and placebo group in a 1:1 ratio. Result measurements will include therapeutic indicators (Clinical Symptom Rating Scale, Phlegm-Blood Stasis Syndrome Scale, and Seattle Angina Questionnaire) and safety indicators (blood routine, urine routine, electrocardiogram, liver function, and kidney function). The clinical data management system (http://www.tcmcec.net/) will be used to collect and manage data. Quality control will be implemented according to good clinical practice.

**Discussion:**

Previous TCM clinical trials have investigated if adding GLDS to standard routine treatment can improve the therapeutic effect in patients with unstable angina pectoris. This study focuses on the safety and efficacy of GLDS on unstable angina pectoris of phlegm-blood stasis type, in order to obtain relevant clinical evidence.

**Trial registration:**

This study is approved by the Ethics Committee of Guang’anmen Hospital of the China Academy of Chinese Medical Sciences (no. 2019–187-KY-02) and is registered with chictr.org (registration number ChiCTR2000031780).

## Abstract 2

Supplemental Digital Content is available in the text

## 1 Introduction

Risk factors of cardiovascular disease are increasing in China, leading to a continuous increase in the number of cardiovascular disease. According to the “China Cardiovascular Disease Report 2018” released in 2019,^[1]^ the prevalence of coronary heart disease continues to rise. The report estimates that 290 million people suffer from cardiovascular diseases and 11 million people suffer from coronary heart disease. Cardiovascular death accounts for the first cause of death in both urban and rural areas. It is responsible for about 45.50% of deaths in rural areas and 43.16% in urban areas. Since 2012, the mortality rate of coronary heart disease has shown an upward trend, and the rapid increase of acute myocardial infarction (AMI) mortality rate is the main reason. Currently, AMI treatment is percutaneous coronary intervention, but the nosocomial fatality rate of ST-Elevation Myocardial Infarction (STEMI) patients has not decreased significantly due to the obvious delay in the patient's treatment and the low total reperfusion treatment rate.^[2]^ At the same time, the treatment of AMI patients occupies a large proportion of public health resources, which has continued to grow at a rate of 29.15% since 2014. In 2016, the total cost of AMI hospitalization was 19.085 billion yuan.^[3]^ Unstable angina (UA) is a group of clinical angina syndromes that belongs to acute coronary syndrome (ACS), including primary exertional angina, advanced exertional angina, resting angina, post-infarct angina, and variable angina. UA is characterized by a progressive increase in angina symptoms, new episodes of rest or nocturnal angina or an extended duration of angina. Due to the unique pathophysiological mechanism and clinical prognosis of UA, patients may develop AMI if not properly treated on time.^[4]^ Treatment of UA has a positive effect on reducing AMI episodes. Since it is difficult to distinguish between UA and NSTEMI, treatment guidelines have unified diagnosis and treatment.^[5]^ In clinical practice, the treatment of UA is usually aimed at solving patients’ current symptoms, which are prone to relapse or even worsen after discharge.

Unstable angina pectoris belongs to the categories of “Xiongbi” and “Xintong” in Chinese medicine. Chinese medicine has a long history in the treatment of heart disease, and there are rich theoretical knowledge and prescriptions regarding the same.^[6–8]^ A study analyzed the syndrome elements of 411 patients with unstable angina pectoris in six hospitals in Beijing, Hubei, and Henan. The results showed that blood stasis accounted for the largest proportion (72.7%), followed by qi deficiency and phlegm turbidity, which accounted for 48.4% and 22.9%, respectively.^[9]^ Stagnation of phlegm and blood stasis are the main symptoms of unstable angina pectoris.^[10–13]^ As a TCM compound, GLDS are composed of *Trichosanthis fructus* such as *Allii macrostemonis bulbus*, *Salviae miltiorrhizae*, *Amomi fructus*, and *Dalbergiae odoriferae lignum*. Previous clinical studies^[14–16]^ showed that GLDS have good clinical efficacy in treating unstable angina pectoris of phlegm-blood stasis type, with the total effective rate as high as 86.2%. GLDS were derived from the famous veteran Chinese medicine of Guang’anmen Hospital combined with the ancient prescriptions and clinical experience of TCM. They were used to treat unstable angina pectoris of coronary heart disease with phlegm and blood stasis syndrome by promoting blood circulation and resolving phlegm method of TCM. GLDS have been used in clinical practice of TCM for more than 60 years, which is also accepted by most Chinese patients. Nevertheless, the clinical safety and efficacy of GLDS in the treatment of unstable angina pectoris patients with phlegm-blood stasis syndrome still lacks accurate verification.

## 2 Methods and analysis

### 2.1 Objective

The objective of this randomized, controlled, double-blind trial is to assess the effectiveness and safety of GLDS in the treatment of unstable angina pectoris.

### 2.2 Trial design

**Figure 1:**
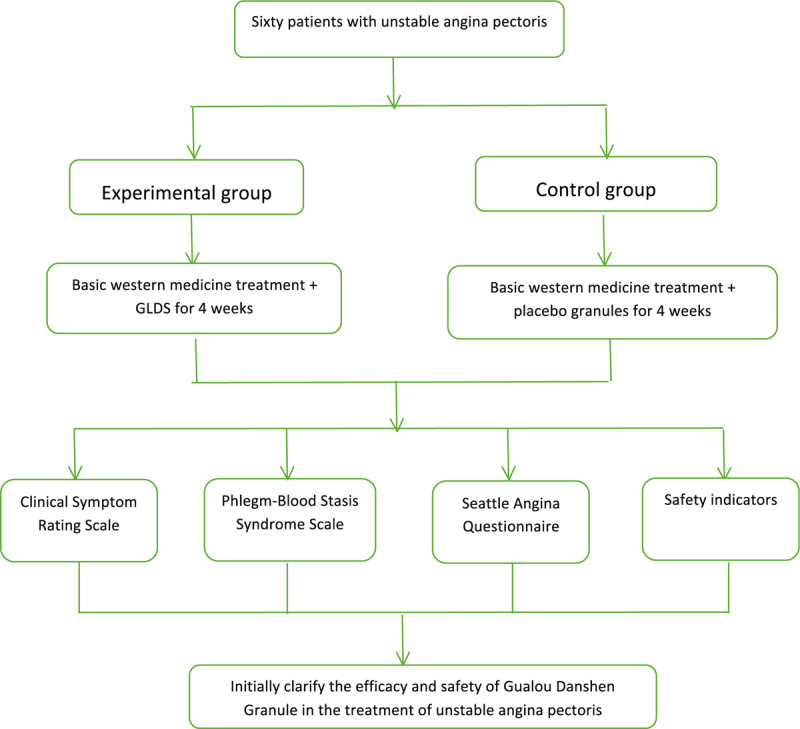
GLDS = Gualou Danshen granules.

Funders will not be involved at any point during the entire process, including research design, data collection, analysis and interpretation, and manuscript writing. Sixty patients will participate in this trial at the Guang’anmen Hospital of the Chinese Academy of Medical Sciences in accordance with the established inclusion and exclusion criteria. All the information about the study will be fully explained to the subjects by the researchers, and the subjects will sign an informed consent from before participation in the study. Any information and privacy of participants will be kept strictly confidential. The conduct of the trial and patient safety will be monitored by an independent data and safety monitoring committee, monitored according to established guidelines and stopped as necessary. The study design is summarized in Figure 1.

### 2.3 Registration

Declaration of Helsinki^[17]^ and Good Clinical Practice (GCP)^[18]^ were the reference criteria for developing this study protocol. The trial has been approved by Research Ethics Committee of the aforementioned hospitals (No.2019-187-KY-01) and registered at chictr.org (registration number ChiCTR2000031780).

### 2.4 Recruitment of participants

The trial will recruit patients with two strategies. One is to put up posters on hospital bulletin boards. The contents include suitable conditions for patients, unsuitable people, the anticipated place and time of treatment, and the contact information of test operators. The other is to recruit outpatient patients in the hospital, through seeking the consent of patients who meet the trial standards, to further complete the recruitment of test patients.

### 2.5 Diagnostic criteria

The criteria for the diagnosis of unstable angina pectoris in this trial were formulated according to the Guidelines for the Diagnosis and Treatment of Unstable Angina Pectoris and Non-ST Segment Elevation Myocardial Infarction (2007)^[4]^ issued by the Cardiovascular Branch of the Chinese Medical Association. The diagnostic criteria of blood stasis of heart and phlegm obstruction of heart refer to Guiding Principles of Clinical Research on New Chinese Medicines in 2002.^[19]^

### 2.6 Inclusion criteria

1. Conforms to the diagnostic criteria of unstable angina pectoris, set out in the guidelines for the diagnosis and treatment of unstable angina pectoris and non-ST-segment elevation myocardial infarction published by the Chinese society of cardiovascular diseases. The diagnostic criteria of TCM syndromes are consistent with phlegm and blood stasis obstruction syndrome.
2. Patients with coronary heart disease confirmed by coronary angiography (1 or more major coronary artery stenosis degree ≥ 50%).
3. Aged 35 to 75 years.
4. Voluntary participation in this clinical study and signed informed consent form. Subjects must take the medicine for 4 weeks, complete the scale and test before and after treatment. A fixed contact way to complete the follow-up work is also requisite.

### 2.7 Exclusion criteria

1. Those who have been confirmed to have chest pain caused by stable angina pectoris, AMI, aortic dissection, other congenital heart disease, valvular disease, severe neurosis, etc.
2. Poorly controlled hypertension (systolic blood pressure ≥160 mm Hg or diastolic blood pressure ≥100 mm Hg), severe cardiopulmonary dysfunction (heart function level III or IV).
3. Chest tightness and pain caused by arrhythmia.
4. Acute stage of cerebral infarction.
5. Patients with severe primary diseases such as liver, kidney, or hematopoietic system disease, or liver function ALT or AST value > 1.5 times the upper limit of normal value, or those with abnormal renal function.
6. Those with an acute infection within the last 2 weeks.
7. Those with other serious diseases that must be treated (such as blood diseases, tumors, etc).
8. Diabetes that does not meet the general control standards, or if more serious diabetes complications occur.
9. Women who are pregnant or breastfeeding.
10. Those with known allergies or allergies to the test drug ingredients.

In addition,

1. patients who are participating in other clinical trials or patients whose clinical trials finished less than 1 month ago will be excluded;
2. if researchers believe that there are other reasons rendering the subject unsuitable for clinical trials.

### 2.8 Randomization and blinding

The randomized grouping method will be used by the clinical evaluation center of the Chinese Academy of Medical Sciences. Random grouping tables will be generated by SAS 6.12 statistical software. The sixty eligible participants will be assigned to either the GLDS group or placebo group in a ratio of 1:1. All drugs will be labeled by pharmaceutical companies according to the random grouping table. Double blindness will be guaranteed during the whole experiment. Random grouping numbers will be stored in opaque sealed envelopes until the end of the experiment or an emergency occurs.

### 2.9 Sample size estimation

According to the calculation formula of sample size, the total effective rate of GLDS is 86.2% in combination with previous clinical study information. Consequently, the test set *a* = 0.05, beta = 0.10, *f* (*a*, beta) = 10.5, using a unilateral test, the initial estimated sample number was 27.5, and the integer number was 28. According to the test funds and other related issues, the drop-out rate was limited to <5%. The number of cases in each group was set at 30, and the total number of cases in two groups at 60.

### 2.10 Intervention

The loading test method will be used. Both groups will be treated with routine western medicine. The routine western medicine treatment is strictly in accordance with the “Guidelines for the Management of Unstable Angina Pectoris-Non-ST Segment Elevation Myocardial Infarction 2012ACCF/AHA.” The experimental group will be given GLDS, 6 g, three times a day. The control group will be given placebo granules, 6 g, three times a day. The placebo is mainly composed of starch, which ensures that the shape and odor of the placebo granules are the same as those of the GLDS. The course of treatment was set for 4 weeks, during which no other Chinese patent medicine or decoction was used to treat coronary heart disease.

### 2.11 Outcomes

Incomplete data will affect the judgment of efficacy and safety, so it can only be adopted when the data integrity is over than 80%, data that does not meet this requirement will be excluded. The data collected will be the following:

1. Clinical Symptom Rating Scale (supplementary file 1);
2. Phlegm-Blood Stasis Syndrome Scale (supplementary file 2);
3. Seattle Angina Questionnaire (supplementary file 3);
4. Chinese medicine syndrome efficacy evaluation criteria in accordance with the Guidance of Clinical Research on New Chinese Medicine for Coronary Heart Disease Angina Pectoris issued by the Ministry of Health of the People's Republic of China in 2002.

The efficacy calculation is as follows: Curative effect index (n) = (pre-test integral-post-test integral)/pre-test integral × 100%. Effectiveness is defined as: significant effect: the clinical symptoms and signs were significantly improved, and the integral of TCM syndromes was reduced by more than 70%. Effective: The clinical symptoms and signs were improved, and the integral of TCM syndromes was reduced by more than 30%, <70%. Invalidity: Clinical symptoms and signs did not improve significantly, or even worsened, the TCM syndrome score decreased by less than 30%. Aggravation: clinical symptoms and signs were aggravated, and the integral of TCM syndromes decreased <0.

All the scales will be completed on the day of registration and after taking the medicine for 4 weeks. We will compare the index changes before and after taking the drug and perform data collation for statistical analysis in order to evaluate the effectiveness of GLDS in the treatment of unstable angina pectoris.

### 2.12 Safety and adverse events

Safety indicators will be recorded before and after 4 weeks of treatment including:

1. vital signs, such as body temperature, blood pressure, respiration, and heart rate;
2. blood routine, urine routine;
3. electrocardiogram;
4. liver function (ALT, AST), renal function (BUN, Cr), etc.

Standard operating procedures for handling all adverse events will be monitored and reported, including occurrence time, severity, duration, effective measures, and results. Investigators will decide whether to suspend or withdraw the trial based on the severity of adverse events. In case of any serious adverse event, the researchers will report it to the principal investigator (Q.H.) and the Medical Ethics Committee within 24 h. Guang’anmen Hospital provided insurance for injuries caused by interventions during the trial period.

### 2.13 Data management and statistical analysis

The general data, safety indicators and therapeutic effect of angina pectoris are to be observed before and after treatment. SPSS 20.0 statistical software will be used for data analysis. The measurement data will be expressed as mean (+standard deviation). One-way analysis of variance will be used for comparison among multiple groups, and the homogeneity of variance test will be conducted using the Levene method. If the variance is homogeneous, LSD-T test will be used for pair comparison. If the variance is not uniform, the Tamhane method will be used for pair comparison. Non-normally distributed data will be represented by median and quartile, using rank sum test. *P* < .05 will be considered statistically significant.

### 2.14 Quality control

To ensure the quality of the experiment, all practitioners, including investigators, research assistants and statisticians, must participate in training and remain unchanged. Researchers will be trained in patient screening, data filling, drug use, adverse event reporting and other matters before the trial. Interventions will be conducted on the basis of strict compliance with standardized operating procedures. The researchers will load the data into the case questionnaire in a timely, complete, correct, and clear manner based on the subjects’ original observation records. After 4 weeks, the investigator shall truthfully record the number of drugs that the subject accepts, takes or returns, determine the subject's medication compliance, and timely record on the case report form.

### 2.15 Patient and public involvement

A pilot trial was conducted before we started this randomized controlled clinical trial. Volunteers were selected from our outpatients to participate in this study. We popularized the main contents of the intervention through education courses, and perfected the deficiencies of the experimental program through the seminars participated by volunteers. To express our gratitude to the volunteers, we also gave each of them an informal report on the results of the study.

## 3 Discussion

Coronary atherosclerotic heart disease (CAD) refers to heart disease caused by coronary atherosclerosis with left main coronary artery stenosis ≥50% and one or several main coronary artery stenosis ≥70%. UA is a type of coronary heart disease. Patients with microvascular dysfunction and coronary artery spasm usually have symptoms related to ischemia or hypoxia.^[20–22]^ Clinical observations have found that the nature of chest discomfort in patients with unstable angina pectoris is similar to that of typical stable angina pectoris, but it is usually more severe and lasts longer. Chest pain can also occur at rest. There is a consensus in Western medicine on the diagnosis and treatment of patients with unstable angina pectoris, such as nitroglycerin for pain relief, aspirin and heparin as anticoagulant therapies, coronary angiography interventional therapy, amongst others.^[23–25]^ However, it is difficult to accurately distinguish between patients with unstable angina pectoris and those with stable angina, and the improvement in symptomatic pain and life management by routine treatment is not significant. Some patients have even developed resistance to aspirin.^[26]^

TCM has a unique system of syndrome differentiation and treatment. It believes that unstable angina pectoris of coronary heart disease belongs to the “Xiongbi” and “Xintong” disease in TCM, and its naming and treatment methods have been known in TCM for thousands of years.^[27]^ According to the syndrome differentiation and description in TCM, unstable angina pectoris with coronary heart disease can be divided into syndromes such as deficiency of qi and Yin, mutual obstruction of phlegm and blood stasis, and coldness of heart vessels.^[28–31]^ The most common clinical syndrome is phlegm-blood stasis, which is very similar to the pathological description of coronary heart disease. Therefore in clinical TCM, the treatment of unstable angina pectoris diagnosed as phlegm-blood stasis is particularly important.

GLDS evolved from traditional prescriptions of TCM. Not only do they have a foundation that is widely circulated among Chinese people in terms of efficacy, but animal experiments of related drugs also provide a preliminary guarantee for the safety of clinical applications. However, the relevant clinical trials that have been conducted lack international standards, and more clinical studies are needed to confirm the applicability and accuracy of this treatment.^[32]^ According to strict clinical design criteria,^[17,18]^ a randomized, controlled, parallel, double-blind clinical study of GLDS is to be conducted in this protocol. However, the design scheme of this experiment still has limitations. The experiment is designed in a single center, ignoring the effect of patients’ geographical origin. In addition, due to the involvement of fund support and related subject cycles, the number of cases included in the design is relatively small, so the next step is still to conduct an in-depth study of large medical records with multiple centers. Therefore, in this standardized preliminary study, we will evaluate the safety of GLDS and its improvement in patients with unstable angina pectoris due to CAD by observing relevant evaluation scales and biomedical parameters.

## Author contributions

Jianbo Guo and Shuang Dai contributed equally to this article and wrote the draft. Qingyong He designed the study. Yukun Ding, Haoqiang He, and Hui Zhang modified the articles. Wenchao Dan, Kun Qin, Hui Wang, and Anqi Li developed the questionnaire draft. Peipei Meng and Shangjin Li participated in statistical analysis. All authors read and approved the final manuscript.

## Supplementary Material

Supplemental Digital Content

## Supplementary Material

Supplemental Digital Content

## Supplementary Material

Supplemental Digital Content
