# Supplementary material for: A randomized controlled trial for gualou danshen granules in the treatment of unstable angina pectoris patients with phlegm-blood stasis syndrome
Source: Medicine (Baltimore). 2020 Aug 14;99(33):e21593. doi: 10.1097/MD.0000000000021593 (PMC7437832; doi:10.1097/MD.0000000000021593)
Supplement: Supplemental Digital Content [file medi-99-e21593-s001.doc]

**(1)Clinical Symptom Rating Scale**

| Angina symptom | | | Score |
| --- | --- | --- | --- |
| Attack number of times    (In the last week) | None | No attacks within a week | □0 |
|  | Light | 2-6 attacks within a week | □2 |
|  | Middle | 1-3 attacks per day | □4 |
|  | Severe | More than 4 attacks per day | □6 |
| The degree of pain | None | No attacks within a week | □0 |
|  | Light | Physical activity heavier than daily activity causes angina | □2 |
|  | Middle | Daily physical activity causes angina pectoris and daily activities are restricted | □4 |
|  | Severe | Physical activity that is lighter than daily activities causes angina, and daily activities are significantly restricted | □6 |
| Time ofduration min  （Maximum duration） | None | No attacks within a week | □0 |
|  | Light | Each pain lasts ≤5 minutes | □2 |
|  | Middle | 5 minutes ＜Each pain lasts ＜10 minutes | □4 |
|  | Severe | Each pain lasts ≥10 minutes | □6 |
| Nitroglycerin  dosage  pills  (In the last week) | None | None | □0 |
|  | Light | Take 1-4 tablets per week | □2 |
|  | Middle | Take 5-9 tablets per week | □4 |
|  | Severe | Take more than 10 tablets per week | □6 |
| Total scores | | |  |
| Nitroglycerin content | | □<0.5mg/d □0.5-2.0mg/d □>2.0mg/d □None | |
| Usage count | | times/ day，per dose： mg，total daily use： mg | |
| Number of times per week | | times/ week，total weekly use： mg | |
