# Supplementary material for: A randomized controlled trial for gualou danshen granules in the treatment of unstable angina pectoris patients with phlegm-blood stasis syndrome
Source: Medicine (Baltimore). 2020 Aug 14;99(33):e21593. doi: 10.1097/MD.0000000000021593 (PMC7437832; doi:10.1097/MD.0000000000021593)
Supplement: Supplemental Digital Content [file medi-99-e21593-s002.doc]

**(2)Phlegm-Blood Stasis Syndrome Scale**

| TCM symptoms | | | Score |
| --- | --- | --- | --- |
| Chest pain | None. | | □0 |
|  | There are more typical chest tingling and cramping, which are fixed and do not move. Each time lasts for a few minutes. The pain occurs at least 2-3 times a week, or 1-3 times a day, but the pain is not serious. Sometimes you need to take nitroglycerin. | | □2 |
|  | There are several typical chest tingles or cramps several times a day, which are fixed and do not move. Each time lasts a few minutes to 10 minutes. The pain is severe. Generally, need to take nitroglycerin. | | □4 |
|  | There are typical chest tingles or cramps several times a day, which are fixed and do not move, thus affecting daily life activities (such as stool, dressing, etc.). Each episode takes a long time and requires multiple doses of nitroglycerin. | | □6 |
| Chest tightness | None | | □0 |
|  | Slight chest tightness. | | □2 |
|  | Chest tightness is obvious, sometimes sigh-like breathing, short of breath after activity. | | □4 |
|  | Chest tightness like suffocation and sighing constantly. | | □6 |
| palpitations | None. | | □0 |
|  | Occasional palpitation, slight discomfort. | | □1 |
|  | Sometimes attacks, lasting a long time, discomfort is obvious. | | □2 |
|  | Often attack, difficult to calm, even affect life. | | □3 |
| Diet | None | Good appetite. | □0 |
|  | Light | Appetite in general. | □1 |
|  | Severe | Poor appetite. | □2 |
| Physically heavy and sleepy | None | | □0 |
|  | Yes | | □1 |
| The lips and tongue are purple and dark  or have ecchymosis | None | | □0 |
|  | Yes | | □1 |
| Sleep | None | Sleep normally. | □0 |
|  | Light | Sleep easy to wake up or sleep instability, wake up early in the morning, but does not affect the work. | □1 |
|  | Middle | Sleep less than 4 hours, but still keep working. | □2 |
|  | Severe | Stay awake all night and have difficulty continuing to work | □3 |
| Total scores | | |  |
