# Supplementary material for: A randomized controlled trial for gualou danshen granules in the treatment of unstable angina pectoris patients with phlegm-blood stasis syndrome
Source: Medicine (Baltimore). 2020 Aug 14;99(33):e21593. doi: 10.1097/MD.0000000000021593 (PMC7437832; doi:10.1097/MD.0000000000021593)
Supplement: Supplemental Digital Content [file medi-99-e21593-s003.doc]

**(3)Seattle Angina Questionnaire**

1.In the past 4 weeks, due to chest pain, chest tightness, and angina, the following restrictions have been imposed:

|  | Severely restricted | Moderately restricted | Slightly restricted | A little bit restricted | Unrestricted | Other |
| --- | --- | --- | --- | --- | --- | --- |
| Dress himself | □1 | □2 | □3 | □4 | □5 | □6 |
| Indoor walking | □1 | □2 | □3 | □4 | □5 | □6 |
| Take a shower | □1 | □2 | □3 | □4 | □5 | □6 |
| Climb a slope or staircase (three floors, without stopping) | □1 | □2 | □3 | □4 | □5 | □6 |
| Do outdoor activities or carry groceries | □1 | □2 | □3 | □4 | □5 | □6 |
| Walk briskly the length of the way (one kilometer) | □1 | □2 | □3 | □4 | □5 | □6 |
| Jogging (one kilometer) | □1 | □2 | □3 | □4 | □5 | □6 |
| Lift or move heavy objects | □1 | □2 | □3 | □4 | □5 | □6 |
| Vigorous exercise (eg swimming) | □1 | □2 | □3 | □4 | □5 | □6 |

2.Compared with 4 weeks ago, during the most intense activities, the occurrence of chest pain, chest tightness and angina:

Significant increase □1 Slight increase □2 Same □3 Slight decrease □4 Significantly decrease □5

3.The average number of episodes of chest pain, chest tightness, and angina during the past 4 weeks:

≥4 times/day □1 1-3 times/day □2 ≥3 times/week □3 1-2 times/week □4 ﹤1 time/week □5 No attack □6

1. In the past 4 weeks, the average number of nitro drugs (such as nitroglycerin) taken for chest pain, chest tightness and angina:

≥4 times/day □1 1-3 times/day □2 ≥3 times/week □3 1-2 times/week □4 ﹤1 time/week □5 Not used □6

5.Troubles caused by taking medicines due to chest pain, chest tightness, and angina:

Severe □1 Moderate □2 Mild □3 Very rarely □4 None □5 Doctor did not administer □6

6.Satisfaction with various measures to treat chest pain, chest tightness and angina:

Not satisfied □1 Mostly dissatisfied □2 Partially satisfied □3 Mostly satisfied □4 Highly satisfied □5

7.Satisfaction with the doctor's explanation of chest pain, chest tightness and angina:

Not satisfied □1 Mostly dissatisfied □2 Partially satisfied □3 Mostly satisfied □4 Highly satisfied □5

8.Overall, satisfaction with the current treatment of chest pain, chest tightness, and angina:

Not satisfied □1 Mostly dissatisfied □2 Partially satisfied □3 Mostly satisfied □4 Highly satisfied □5

9.In the past 4 weeks, chest pain, chest tightness, and angina have affected the degree of fun in life:

Not satisfied □1 Mostly dissatisfied □2 Partially satisfied □3 Mostly satisfied □4 Highly satisfied □5

10.How will you feel if you have chest pain, chest tightness, and angina in your future life:

Not satisfied □1 Mostly dissatisfied □2 Partially satisfied □3 Mostly satisfied □4 Highly satisfied □5

11.Worry about heart attack and sudden death:

Always worried □1 Frequently worried □2 Sometimes worried □3 Few worried □4 Never worried □5
